# Oral Rehabilitation with Removable Partial Denture of a Patient with Cleidocranial Dysplasia

**DOI:** 10.1155/2020/8625842

**Published:** 2020-05-09

**Authors:** Ali Jamali Ghomi, Reza Sayyad Soufdoost, Mohammad Saeed Barzegar, Mohammad Ali Hemmati

**Affiliations:** Department of Prosthodontics, Faculty of Dentistry, Shahed University, Tehran, Iran

## Abstract

This case report describes the oral rehabilitation of a patient with cleidocranial dysplasia who received a removable partial denture along with silicone-based permanent soft liner to improve esthetic and masticatory function. This patient was the candidate of neither implant nor orthodontic treatment due to medical conditions, history of mandible fracture, age, and risk of fracture after mandibular teeth extractions. Cone-beam computed tomography has made it possible to obtain comprehensive information regarding the morphology and positional relationship of impacted supernumerary teeth. Also, proper collaboration between surgeon and prosthodontist helped to achieve significant improvements in patient's self-esteem, masticatory function, and esthetic.

## 1. Introduction

Cleidocranial dysplasia is a hereditary congenital disorder which results in generalized skeletal dysplasia [1]. The RUNX2 gene mutations that cause cleidocranial dysplasia reduce or eliminate the RUNX2 protein activity in each cell, decreasing the total amount of functional RUNX2 protein. This lack of functional RUNX2 protein can affect the normal development of bones, cartilage, and teeth, resulting in the signs and symptoms of cleidocranial dysplasia [2]. It is characterized by prominent parietal, frontal and occipital bone, underdeveloped paranasal sinus, supernumerary teeth, short shoulder blades (scapulae), an abnormal curvature of the spine (scoliosis), wide-set eyes (hypertelorism), a flat nose, a small upper jaw, and many other abnormalities [1–4].

General dentists can easily diagnose the CCD through a panoramic radiograph taken in addition to other clinical findings [3]. Phenotypic characteristics in the oral cavity include the presence of impacted teeth which could be diagnosed as supernumerary teeth, delayed eruption of permanent teeth, and prolonged retention of primary teeth [4]. Tsuji et al. [5] have reported the average number of supernumerary teeth 7.8 and the average number of unerupted permanent teeth 17.8, in CCD patients aged 15 to 25 years old. In general, supernumerary teeth occur in 6% or more of the normal population [5].

Cone-beam computed tomography (CBCT) has been introduced as the most recent advancement in maxillofacial imaging, enables clinicians to view the morphology of the skull and the dentition in all three dimensions and to design the best treatment plan [6]. The advantages of CBCT over 2D images make it the ideal tool for probing and managing oral and maxillofacial defects [7]. In a patient with CCD, the 3D views by CBCT for evaluation of unerupted, impacted, or supernumerary teeth and their association with vital structures have been essential and inevitable [8].

The treatment plan largely depends on the age [9] and a patient's medical condition [10]. On the other hand, esthetic and masticatory functions are two essential needs, which should be provided by therapists [5]. Dental treatment in the patients with CCD requires an interdisciplinary approach involving orthodontists, maxillofacial surgeons, and prosthodontists [11]. Therapeutic options are varied from the extraction of all teeth followed by the fabrication of denture to implant placement and orthodontic treatment [1]. However, there appears to be a trend in favor of the use of the implant-supported prosthesis instead of conventional removable partial dentures [12].

Over the years, many modifications have been performed in the designing of the complete dentures, but still in compromised cases, residual ridge may be subjected to trauma by the rigid denture base which is in close contact with the soft tissues [13]. Long-term soft denture lining (LTSDL) materials constitute a group of polymer materials using to modifying the trauma associated with wearing complete dentures. They can remain in the oral cavity for at least four weeks. However, their use can extend to several months or even years practically [14]. They reduce the traumatic effect that a denture may have on patients with thin atrophic mucosa, ridge atrophy or resorption, deep anatomical undercuts, bruxism tendencies, or where the oral mucosa exhibits a reduced tolerance to the load applied by a denture, and in congenital and acquired oral defects requiring repair [13, 15]. Soft denture lining materials lead to a more uniform distribution of stress at the mucosa/lining interface, which results in that wearing the complete prosthesis becomes more comfortable for the patient [14]. Currently, two types of soft denture lining material are available: silicone elastomers and soft acrylic compounds. In the clinical situation, the silicone materials are preferred because they remain more stable, while the acrylic materials undergo a more marked loss of cushioning effect over time [14].

## 2. Case Report

**Figure 1:**
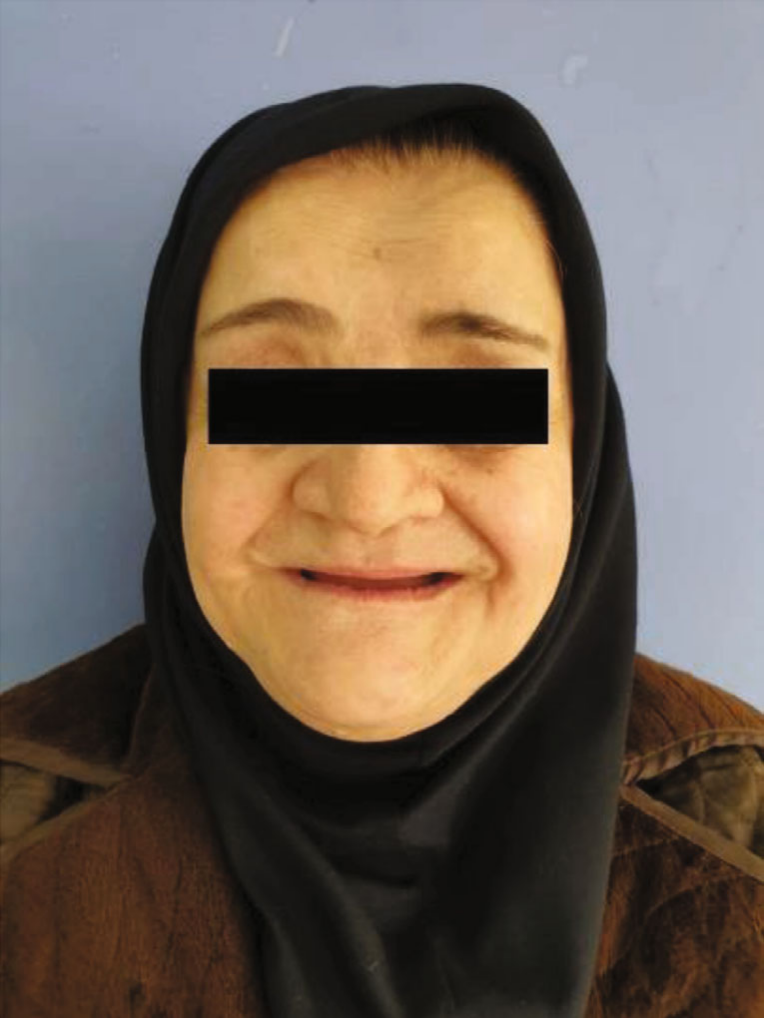
Mandibular prognathism with depressed nasal bridge and frontal prominence.

**Figure 2:**
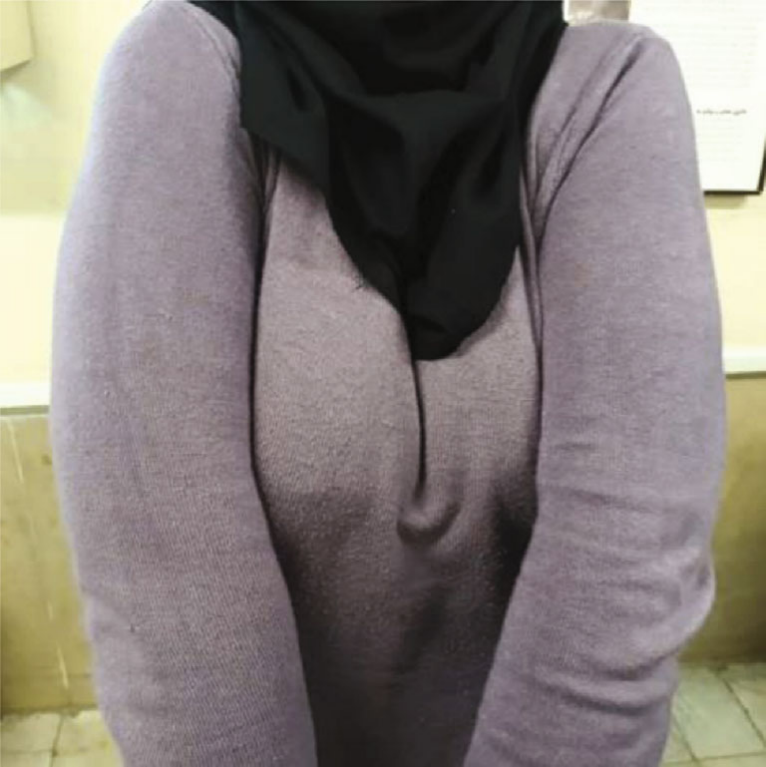
Approximation of shoulders in front of the chest.

A 60-year-old female came to the diagnosis of the oral disease department of Shahed Dental School for dental treatment with a chief complaint of lack of masticatory capability and poor esthetic for the past 20 years. Her medical history was significant for osteoporosis and congenital heart disease (CHD). Her current medication was the daily use of ASA as an antithrombotic, Digoxin for CHD, Enalapril for her hypertension, and vit D. Also, she had been medicating with Famotidine and Omeprazole for the last two years due to her masticatory hypofunction and digestive problems. Her medical practitioner advised that her dental treatment should be limited to noninvasive dental procedures with minimal trauma. The patient revealed that there was an absence of the lower and upper anterior teeth after exfoliation of deciduous teeth and the history of mandibular fractures 25 years ago in a motor vehicle collision. In extraoral examination, the prominent frontal and parietal, hypertelorism, depressed nasal bridge, mandibular prognathism, and maxillary hypoplasia were observed (Figure 1). Also, the patient was able to move shoulders in front of the chest associated with underdeveloped or absent collarbone (Figure 2).

**Figure 3:**
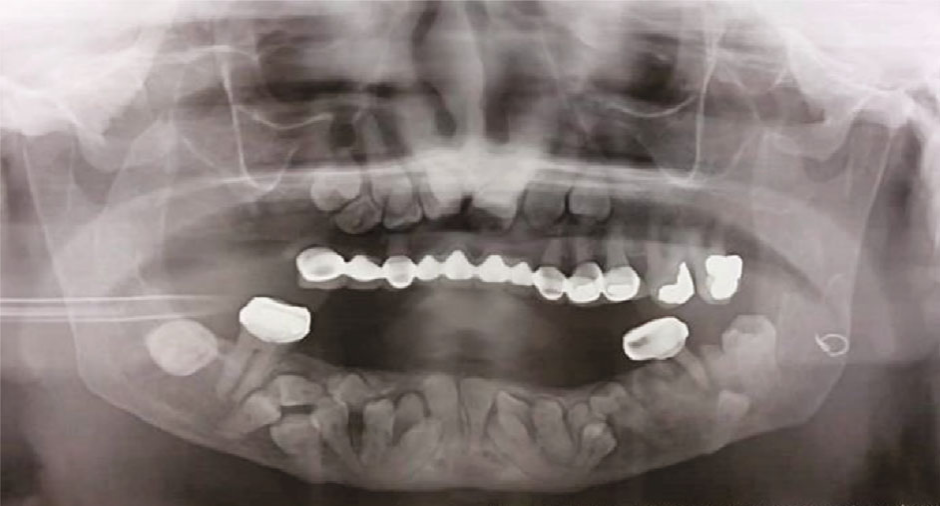
Panoramic radiograph before treatment.

**Figure 4:**
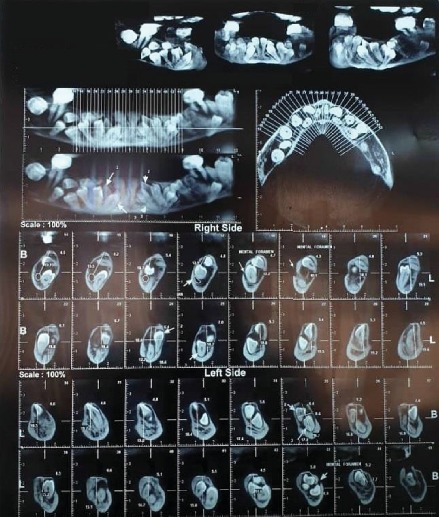
CBCT revealed 21 impacted teeth in mandible and 9 in maxilla.

Intraoral examination revealed a long span bridge in maxilla from tooth #15 to #25 and multiple missing permanent teeth in the anterior and posterior regions of the mandible. A panoramic radiograph and CBCT were captured (Figures 3 and 4).

**Figure 5:**
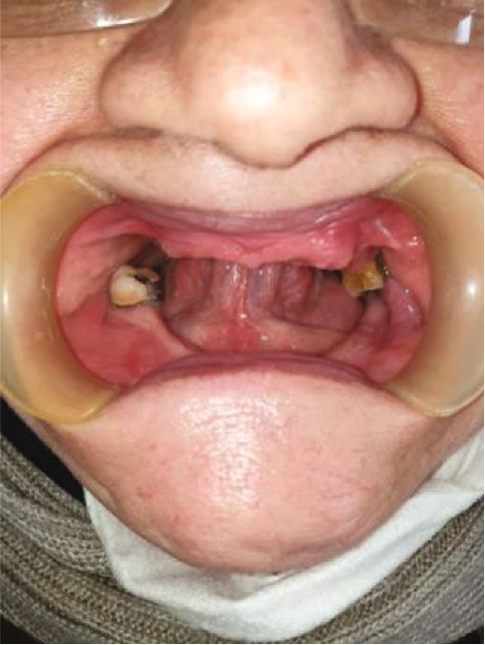
Intraoral photograph before prosthodontic treatment.

Radiographic findings revealed multiple impacted permanent teeth in the anterior region of maxilla and anterior and posterior regions of the mandible as well as the fracture of the left angle of the mandible which had been fixed with wire. The patient was diagnosed with cleidocranial dysplasia evidenced by clinical and radiographic findings. After consulting with a maxillofacial surgeon, the extraction of all teeth (erupted and unerupted) and replacement by implants was not suggested due to the medical conditions of the patient including osteoporosis and CHD. The most conservative and minimally invasive treatment recommended by the surgeon. Therefore, removable partial denture (RPD) was planned to restore both arches. The situation was explained to the patient in detail. A treatment plan including RPD after extraction of hopeless teeth was chosen by the patient, which was the same to therapeutic team choice. The long span maxillary bridge was removed and the tooth #15, 13, 23, 24, 25, 36, and 38 were diagnosed hopeless. The extraction of these teeth was performed under local anesthesia and with minimal trauma. Socket preservation was done with bone graft materials to assist regeneration and healing, and to avoid severe bone loss, particularly in the site of tooth #38. On the other hand, the tooth #26, 27, and 46 were saved due to their stable conditions, increasing the retention and support of the removable partial prosthesis (Figure 5).

**Figure 6:**
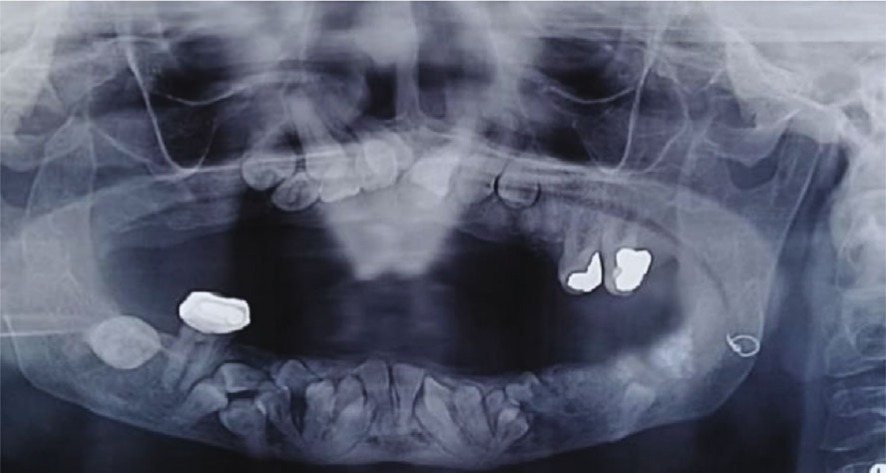
Panoramic radiograph 3 months after teeth extraction.

After 3 months, a new OPG was captured, which showed no significant mandibular and maxillary bone resorption (Figure 6).

**Figure 7:**
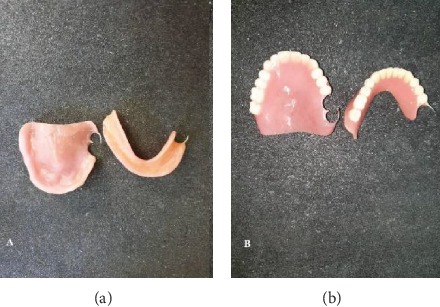
Interior (a) and exterior (b) surfaces of maxillary and mandibular removable partial denture.

**Figure 8:**
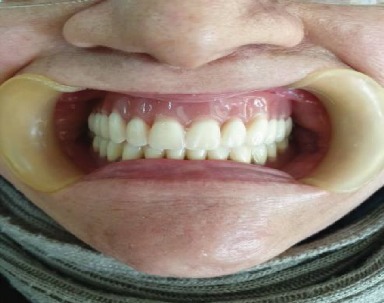
Final oral rehabilitation with removable partial denture.

Preliminary impressions were made for both arches using irreversible hydrocolloid impression material (CA37; Cavex Holland BV, Haarlem, Netherlands) in a stock tray. Impressions of both arches were poured to obtain study casts by dental plaster type 2 (Pars Dandan, Iran). Acrylic custom trays were made using auto polymerizing acrylic resin (Bisico-Germany). In the second visit, both custom trays were border molded using green stick compound, the definitive impression was recorded with Panasil initial contact light impression material (Kettenbach, Germany), and final casts were poured in type 3 dental stone (Pars Dandan–Iran). Acrylic resin base and wax rims were fabricated, and maxillomandibular relationships were recorded by facebow. Maxillary and mandibular diagnostic tooth arrangements were prepared to evaluate phonetic and esthetic, teeth position, and to create maxillomandibular relationship. Afterwards, acrylic resin partial dental prostheses were processed from heat-polymerized acrylic resin (Kulzer, Germany) with a heat-cured permanent silicone soft liner (silicone based detax, Germany) and delivered (Figures 7(a) and 7(b) and 8).

**Figure 9:**
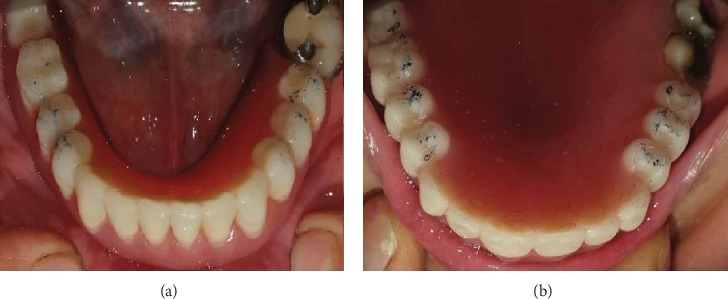
Final adjustment of mandibular (a) and maxillary (b) prostheses.

Wrought wire retentive arm was designed on tooth #27 and 46 to elevate the retention of partial dentures. In order to minimize the microbial/fungal colonization of liners and prolong their life, the patient was trained in two sessions on how to maintain the oral and denture hygiene in good condition. Also, the patient was advised to use prostheses except when asleep. An appointment was scheduled after a week for the final adjustment (Figures 9(a) and 9(b)).

The patient was examined clinically and radiographically every 3 months, throughout a year. Her quality of life and mastication function was improved significantly, and the patient was satisfied. The prognosis of the mandibular and maxillary natural molars tooth #26, 27, and 46 were excellent. The maxillary and mandibular partial denture did not need to be relined after a year.

## 3. Discussion

The treatment of a patient with CCD can be more challenging and complicated, particularly in older adults [16]. To achieve the best treatment plan and satisfaction of patients with CCD, an interdisciplinary dental approach plays an important role [1]. Prosthodontist, orthodontist, and oral surgeon should be involved in creating a comprehensive treatment plan for a patient with CCD [11]. Tailored combination of surgery, orthodontics, and prosthodontics is necessary to provide a functional dentition and reconstruct the smile and facial contour of patients with CCD [1]. The dental treatment in CCD varies and primarily depends on patient's needs, medical conditions, social and economic circumstances, and age of diagnosis, but still the main purpose of treatment is to improve craniofacial and dental function together with aesthetic [5].

Early diagnosis of CCD is very imperative so as to design the best treatment plan and treatment duration [17]. The premature diagnosis leads to a proper orientation for the treatment [16]. There is no consistent protocol for patients with CCD who seek treatment at different ages [1]. In some cases [1, 10, 18], the orthodontic treatment was suggested after the extraction of retained deciduous teeth. In these cases, subsequent orthodontic alignment resulted in a functional and esthetic outcome, and a good facial profile. However, most of these papers just focused on early operation at age 6-12. In our case, orthodontic treatment was not possible with respect to age (60 years), medical conditions, and time of diagnosis. However, if it had been assessed in the earlier age, the orthodontic treatment might have been possible by the extraction of retained deciduous and supernumerary teeth with surgical exposure of impacted permanent teeth and orthodontically guided eruption [17].

In many recent cases, the extractions of impacted teeth followed by implant replacement were suggested as the best treatment plan for patients with CCD [16–18]. In the present case, possible treatment alternatives were discussed with the maxillofacial surgeon with respect to systemic problems, medications, age, and patient's demands. CBCT revealed the numerous impacted teeth in the maxilla (9) and mandible (21), near the inferior border of the mandible, demonstrating the fracture risk of the remaining bone, and damage to the inferior alveolar nerve after extraction. Also, age and osteoporosis were two important factors influencing bone healing after extraction. Petropoulos et al. [19] reported that a genetic defect in patient with CCD may negatively affect the osteoblastic activity around implants and subsequently results in the weaker osseointegration. Considering the above reasons and the history of fracture in angle of the mandible in the left side, treatment planning of full extraction and rehabilitation with implants was ruled out by the surgeon and prosthodontist, despite being a viable alternative.

Some authors suggest that the removal of primary or supernumerary teeth does not promote the eruption of impacted permanent teeth [20, 21]. The lack of cellular cementum is considered to be one of the factors responsible for unerupted teeth in CCD patients [20]. On the other hand, alkaline phosphatase activity has been demonstrated to be consistently reduced in patients with CCD which is another factor associated with delayed eruption in CCD patients [21]. Therefore, it seems that future eruption of the retained teeth in the present case is unlikely. Also, in the present case, maintaining the retained supernumerary teeth could be beneficial for the prevention of more complicated surgeries, severe bone loss, and damage to vital structures [16]. The patient had a history of mandibular fracture, osteoporosis, and systemic problems. These predisposing factors might have raised the risk of mandibular fracture, much bone loss, and psychological trauma in the case of surgery [17].

The preservation of even a single healthy tooth in the oral cavity can stabilize an otherwise unstable denture, have positive effects on the patient's self-esteem, and preserve proprioception and occlusal relationship [16]. Therefore, it was decided to save the tooth number 26, 27, and 46 regarding their stable conditions. In some areas of maxilla and mandible, the mucosa over the embedded teeth was thin, and further resorption expected after wearing the RPD, which might continue to expose the retained teeth. In order to minimize the resorption of residual ridge and mucosa under the rigid base of RPD, silicon-based permanent soft liner was applied. The application of a soft material is intended to increase the comfort of denture wearers and to support prosthetic treatment [22]. The use of LTSDLs is mostly suggested in edentulous patients with ridge atrophy or resorption, bony undercuts, bruxing tendencies, and congenital or acquired oral defects [15]. Also, silicone-based LTSDL materials, which are characterized by more stable hardness, sorption, and solubility than acrylic-based LTSDLs, have been recommended in many studies [14, 15]. As CCD is the rare inherent abnormality, and there is a lack of evidence that what will happen in the future to unerupted teeth, the consistent follow-up is mandatory to monitor patient's conditions [19]. The most challenging aspect in the use of the soft liner was its tendency to support microorganism growth despite the fact that the patient was trained on how to clean and disinfect the denture base in two sessions [14]. In order to solve this problem, and as impacted teeth were not extracted, regular follow-ups were essential to monitor the patient's condition. The patient was examined clinically and radiographically every 3 months for a year.

In the current situation, this case report highlights the importance of this fact that the best treatment is not essentially the complicated one, in contrast, in some cases like our case, noninvasive procedures and realistic expectations are considered as successful treatment, ensuring patient's needs are met. However, long-term follow-ups and more clinical reports are needed to determine the ideal therapeutic approach for CCD patients.

## 4. Conclusion

Dentists should recognize that the more complicated the proposed treatment plan, the less likely the chance of success. In this case report, functional and esthetic rehabilitation was achieved, using RPD in the maxilla and mandible. The minimally invasive procedure was planned regarding the limitations of using implant replacement and orthodontic treatment.
